# LED Lights Affecting Morphogenesis and Isosteroidal Alkaloid Contents in *Fritillaria cirrhosa* D. Don—An Important Chinese Medicinal Herb

**DOI:** 10.3390/plants9101351

**Published:** 2020-10-13

**Authors:** Chia-Chen Chen, Maw-Rong Lee, Chi-Rei Wu, Hsin-Ju Ke, Hui-Min Xie, Hsin-Sheng Tsay, Dinesh Chandra Agrawal, Hung-Chi Chang

**Affiliations:** 1 Department of Chinese Pharmaceutical Sciences and Chinese Medicine Resources, China Medical University, Taichung 40402, Taiwan; ferny1010@yahoo.com.tw (C.-C.C.); crw@mail.cmu.edu.tw (C.-R.W.); 2 Department of Chemistry, National Chung-Hsing University, Taichung 40227, Taiwan; mrlee@dragon.nchu.edu.tw (M.-R.L.); qaznancy@gmail.com (H.-J.K.); 3 Nin Jiom Pharmaceutical Co. Ltd., Taipei 108024, Taiwan; patricia@ninjiom.com.tw; 4 Department of Applied Chemistry, Chaoyang University of Technology, Taichung 41349, Taiwan; hstsay@cyut.edu.tw; 5 Department of Golden-Ager Industry Management, Chaoyang University of Technology, Taichung 41349, Taiwan

**Keywords:** *Fritillaria cirrhosa* D. Don, alkaloid content, callus, in vitro culture, LED lights, light intensity

## Abstract

Investigations were carried out to study the effects of light-emitting diode (LED) lights on growth and development of isosteroidal alkaloids in embryogenic calli of *Fritillaria cirrhosa* D. Don, an important traditional Chinese medicine herb. Calli were cultured in glass bottles, each containing 100 mL of Murashige and Skoog’s basal medium supplemented with 2% sucrose and 0.4% gellan gum powder, a gelling agent. These bottles were incubated in a specially designed plant growth chamber equipped with eight different LED lights consisting of single or combinations of four different light spectra emitting blue (450 nm), green (525 nm), red (660 nm), and far-red (730 nm) light. After three months of incubation, morphological changes in embryogenic calli were recorded, and LC-MS/MS analysis of cultures was carried out for peimisine, sipeimine, peiminine, and peimine. The highest number of somatic embryos and the maximum fresh weight was recorded in calli incubated under red (9R), infrared (9IR), and a combination of red+blue+infrared (3R3B3IR), respectively, in decreasing order. The highest contents of peimisine, peiminine, and peimine were recorded under red (9R) and infrared (9IR) lights, respectively. Eight LED lights had significant effects on the morphogenesis of embryogenic calli of *F. cirrhosa* D. Don and contents of isosteroidal alkaloids.

## 1. Introduction

*Fritillaria,* a bulbiferous and perennial monocot plant genus, belongs to the family Liliaceae. The genus consists of about 130 species distributed in the temperate regions of Central Asia and the Mediterranean region [1]. Though some *Fritillaria* species are grown as ornamental plants, several *Fritillaria* species possess valuable medicinal properties. *Fritillaria* bulbs composed of fleshy, farinaceous scales constitute essential plant parts and have been used to relieve cough for centuries [2]. 

In different *Fritillaria* species, a majority of bioactive compounds (86%) identified so far (~130) consist of isosteroidal alkaloid skeletons [3]. Alkaloids in *Fritillaria* bulbs are the main bioactive compounds responsible for relief from coughs [3,4]. However, the quantities and types of alkaloids vary depending on species [1,3,4,5]. 

In a recent study, it was found that peimine, an alkaloid from *Fritillaria,* blocked the Nav1.7 ion channel and inhibited the Kv1.3 ion channel in HEK 293 cell lines, indicating that the compound has a role in relieving pain and possesses anti-inflammatory properties [6]. More recently, Liu and co-workers investigated the potential effect and mechanism of six isosteroidal alkaloids on oxidative stress. The findings showed that *F. cirrhosa* D. Don bulbs might play a protective role in cellular oxidative stress by activating the Nrf2-mediated antioxidant pathway [7]. 

China is the center of diversity of the *Fritillaria* genus. *F. cirrhosa* D. Don (FC) is an important traditional Chinese medicine commonly known as “Chuanbeimu” (川貝母), and is one of the most exploited species. Due to scarcity, the price of wild *F. cirrhosa* D. Don bulbs escalated almost nine times from $60 to $560 USD between 2002–2017 [8]. Due to the excessive collection of FC bulbs from natural habitats, the species is now under protection [9]. Therefore, alternative propagation methods of FC bulbs and the production of critical isosteroidal alkaloids by tissue culture techniques must be optimized. 

The culture of plant tissues and organs is an important bio-technique to produce secondary plant metabolites under controlled environmental conditions in a laboratory. Under culture conditions, callus (an undifferentiated mass of cells) can easily be induced, practically from any living plant part. Induced callus can be cultured and multiplied in vitro on a defined nutrient medium with or without plant growth regulators. There are several advantages of using callus cultures as a source of valuable secondary metabolites, including (i) ease of induction and multiplication; (ii) production of bioactive compounds throughout the year independent of season; (iii) whole plants do not need to be cultivated, especially rare and endangered species; (iv) amenability of scaling by bioreactors for mass production, etc. More recently, we have reported on the micropropagation of bulblets and the production of isosteroidal alkaloids in tissue culture-derived materials of *F. cirrhosa* D. Don [10]. Several recent studies have demonstrated that the light quality not only affects morphogenetic responses in plants but has significant effects on their physiological processes, including metabolic pathways and the production of secondary metabolites [11,12]. 

A recent review listed several studies on the effects of light quality on the production of secondary metabolites in different plant species [11]. In the present study, four isosteroidal alkaloids (peimisine, sipeimine, peiminine, and peimine) were analyzed considering their therapeutic effects. Peiminine (Pm) is one of the major isosteroidal alkaloids in *Fritillaria* that is reported to have extensive pharmacological activities, including anti-inflammatory [6], anti-cancer [13], and antioxidant [14] capabilities. In another report, sipeimine and peiminine from bulbs of *F. wabuensis* inhibited pro-inflammatory mediators in lipopolysaccharide (LPS) stimulated RAW 264.7 macrophage cells [15]. More recently, it was demonstrated that peimine relieved inflammatory effects in IL-1β-induced chondrocytes, indicating that peimine might be a potential therapeutic agent for osteoarthritis [16]. Antitussive, expectorant, and anti-inflammatory effects of several alkaloids, including sipeimine, chuanbeinone, peiminine, and peimine isolated from Bulbus *Fritillaria cirrhosa* were demonstrated in mice by using a phytochemical method [17]. A most recent study confirmed the anti-cancer effects of sipeimine obtained from bulbs of *F. cirrhosa* against non-small cell lung cancer (NSCLC) both in vivo and in vitro [18]. This anti-cancer property of sipeimine is largely due to anti-inflammation action affected by NF-κB inhibition, making it a potential drug candidate for treating cancer at early stages [18]. Recently, Yin and co-workers have reported several therapeutic properties of peimine, including anti-cancer, anti-inflammatory, antitussive, expectorant, and sedative [19]. A more recent study has also demonstrated cough relief by peimine by affecting the systemic network of proteins and pathways [20].

The objective of the present study is to investigate the effects of different LED lights on growth and development in embryogenic calli and the contents of four isosteroidal alkaloids (peimisine, sipeimine, peiminine, and peimine) in in vitro cultures of *F. cirrhosa* D. Don. Findings in the study may be of help to produce certain alkaloids under laboratory conditions irrespective of the season and thus avoid having to collect *F. cirrhosa* D. Don bulbs from the wild. 

## 2. Material and Methods 

### 2.1. Callus Multiplication

Callus obtained in our previous experiments, as reported earlier [10], was further multiplied in the liquid medium. Callus was cultured in 125 mL Erlenmeyer flasks, each containing 20 mL basal salts and vitamins of Murashige and Skoog [21] medium (MSBM) supplemented with 2,4-D (0.5 mg/L) and 2% sucrose (Sigma). The culture flasks were placed on an orbital shaker (Model SK-302A, Sun Kaun Instruments Co., Taichung, Taiwan) set at 100 rpm and incubated at 25 ± 1 °C in the dark.

### 2.2. Influence of Different Light Spectra on Morphogenesis in Embryogenic Calli and Contents of Isosteroidal Alkaloids

**Figure 1:**
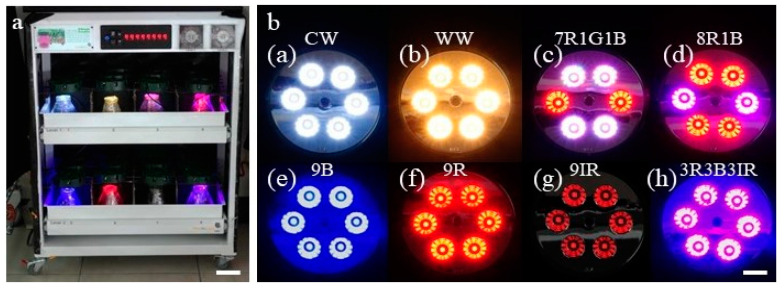
(**a**) Plant growth chamber with LED lights. Bar = 8.3 cm; (**b**) Eight different LED lights: (**a**) CW-5000 K, (**b**) WW-2700 K, (**c**) 7R1G1B, (**d**) 8R1B, (**e**) 9B, (**f**) 9R, (**g**) 9IR, (**h**) 3R3B3IR. Bar = 2 cm. Red (R): 660 nm; green (G): 525 nm; blue (B): 450 nm; infrared (IR): 730 nm.

To investigate the effects of LED lights on the morphogenesis of embryogenic calli and the contents of isosteroidal alkaloids, embryogenic calli from liquid cultures were taken out and kept for 1 min on sterilized filter paper in a laminar flow before inoculation to glass bottles. Callus (3.0 g) was cultured in glass bottles (650 mL capacity), each containing 100 mL of MSBM medium with 2% sucrose and 0.4% gellan gum powder (GPP), a gelling agent (PhytoTechnology Laboratories®, USA). The pH of the medium was adjusted to 5.7 ± 0.1 before the addition of GPP and autoclaving at 1.05 kg/cm for 15 min. To facilitate LED light exposure to cultures, each bottle was closed with a piece of transparent, autoclavable plastic sheet. These bottles were incubated in a specially designed plant growth chamber equipped with eight different LED lights (Nano Bio Light Technology Co., Ltd., Taiwan). The chamber had two tiers, and each tier had four partitions (Figure 1a). Culture bottles were kept in these eight sections and exposed to different light spectra by eight specially designed LED lids (CW-5000K, WW-2700K, 8R1B, 7R1G1B, 3R3B3IR, 6R, 6B, and 6IR) (Figure 1b). LED lids CW-5000K and WW-2700K represented cool (C) and warm (W) white (W) light, while 5000K and 2700K represented color temperature, respectively. As reported in a previous study from our laboratory [22], six of these LED lids emitted single or combinations of four different light spectra with wavelengths such as blue (450 nm), green (525 nm), red (660 nm), and far-red (730 nm). The symbols in each LED lid code and the spectral distribution (quantum ratio) in eight LED lids were as follows: blue (B), green (G), red (R), infrared (IR), CW-5000K (28:43:29:0), WW-2700K (8:46:46:0), 8R1B (16:0:84:0), 7R1G1B (17:9:74:0), 3R3B3IR (57:0:43:37), 9R (0:0:100:0), 9B (100:0:0:0), and 9IR (0:0:0:100). In this work, the number (9, 7, 3, 1) in each LED lid code represents the number of LED chips in a particular lid. The light intensity of each LED lid was as follows: CW-5000K* (57 μmol m^−2^ s^−1^); WW-2700K (56 μmol m^−2^ s^−1^); 7R1G1B (56 μmol m^−2^ s^−1^); 8R1B (57 μmol m^−2^ s^−1^); 9B (57 μmol m^−2^ s^−1^); 9R (56); 9IR (10 μmol m^−2^ s^−1^); 3R3B3IR (56 μmol m^−2^ s^−1^).

The LED growth chamber was set on a 16 h light and 8 h dark cycle, kept in a culture room set at 25 ± 2 °C, and fully covered by a thick, dark cloth to cut off outside light. Morphological features of embryogenic callus under each light condition were recorded after three months of incubation. In addition, cultures under different LED lights were analyzed by LC-MS/MS for contents of peimisine, sipeimine, peiminine, and peimine. 

### 2.3. Development of Bulblets from Somatic Embryos

For the development of bulblets, single somatic embryos (SEs), clusters with five somatic embryos each, and a single embryo with cotyledonary leaf obtained from cultures under CW5000K or WW2700K lights were further transferred to fresh MSBM medium in glass bottles as described in Section 2.2. Each bottle contained seven explants. These bottles were incubated in a culture room with a temperature set at 25 ± 2 °C, and under light (white fluorescent tubes, illumination intensity of 34 μmol m^−2^ s^−1^) with a 16 h light and 8 h dark cycle. Observations of bulblet development in all three types of SEs were recorded after three months of incubation. 

Microphotographs of cultures were taken by a digital camera (Nikon D90, Tokyo, Japan). For SEM, samples of bulblets were frozen in liquid nitrogen and observed using a scanning electron microscope (JEOL JSM-6330F, Tokyo, Japan).

### 2.4. Chemicals and Other Materials

Peimisine, sipeimine, peiminine, and peimine standards were procured from SunHank Technology Co., Ltd., Taiwan. Ammonium formate, sodium acetate, and octadecyltrimethoxysilane were purchased from Sigma-Aldrich (St. Louis, MO, USA). Ammonium hydroxide solution, ferric chloride hexahydrate, ethylene glycol, and acetonitrile were obtained from Fluka (Steinheim, Germany), Alfa Aesar (Heysham, UK), Acros Organics (Morris County, NJ, USA), and J. T. Baker (Phillipsburg, NJ, USA), respectively. Methanol and ethanol were purchased from Merck (Darmstadt, Germany). Ultrapure water (18.2 MΩ) was freshly obtained from a Millipore Simplicity system (MilliporeSigma, Bedford, MA, USA). The stock solutions (1 mg/mL) of each analyte were prepared in methanol separately and stored in the dark at −30 °C. The working solutions were prepared by diluting the stock solutions before use.

### 2.5. Preparation of Fe_3_O_4_@C_18_ Nanoparticle Composite

The adsorbent (magnetic nanoparticles, Fe_3_O_4_@C18) used for magnetic solid-phase extraction was synthesized according to the literature [23,24] with minor modification. Briefly, ferric chloride hexahydrate (2.7 g) and sodium acetate (7.2 g) were dissolved entirely in ethylene glycol (100 mL) before being poured into a Teflon-lined stainless steel autoclave heated at 200 °C for 8 h. The resultant Fe_3_O_4_ nanoparticles were washed with ethanol and dried. Afterward, the Fe_3_O_4_ nanoparticles (10 mg) were dispersed in a mixture of 970 µL ethanol, 10 µL water, and 20 µL octadecyltrimethoxysilane through sonication for 30 s, followed by shaking for 8 h at 45 °C. The derivatized Fe_3_O_4_@C18 was washed with ethanol, water, and acetonitrile three times, respectively. The final product was resuspended in 1 mL of acetonitrile (10 mg/mL). 

### 2.6. Extraction Procedure

Ultra-pure water (2 mL) was added to each 0.1 g powdered sample of in vitro culture, 3-month-old in vitro derived bulblets, and 3-year-old wild type commercial bulbs before vortexing for 1 min and letting stand for 5 min. After centrifugation at 4000 rpm for 5 min, 0.8 mL of supernatant was collected. Then, 8 μL internal standard (50 μg/mL), 50 μL ammonia solution, and 140 μL Fe_3_O_4_@C18 were added into the supernatant with a vortex treatment for 30 s then left to stand still for 5 min. With the help of an external magnet, the analyte-adsorbed Fe_3_O_4_@C18 was rapidly separated from the supernatant and vortexed with 100 μL acetonitrile for 30 s to elute the analytes. After that, the external magnet was used to settle the magnetic adsorbent while the elution solution was filtered through a 0.22 μm PTFE (polytetrafluoroethylene) membrane for LC-MS/MS analysis.

### 2.7. LC-MS/MS Conditions

**Figure 2:**
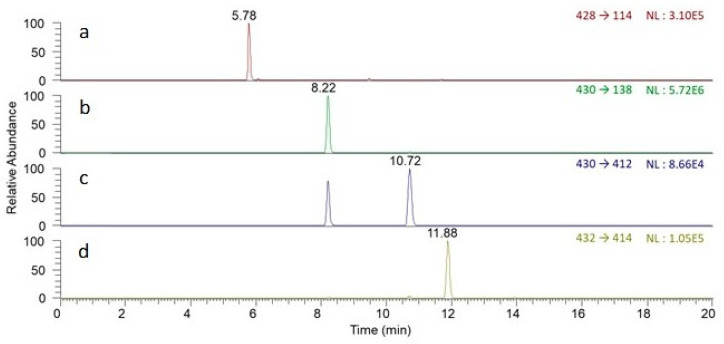
Extracted ion chromatograms of isosteroidal alkaloid standards (0.5 µg/mL): (**a**) Peimisine, (**b**) Sipeimine, (**c**) Peiminine, and (**d**) Peimine.

The analysis was performed with a Surveyor LC-MS/MS system (Thermo Scientific, Waltham, CA, USA). The chromatographic separation was achieved by using a Thermo Scientific Accucore C18 column (2.1 × 100 mm, 2.6 µm) at a constant column temperature of 30 °C with a flow rate of 0.2 mL/min. The mobile phase A was 10 mM ammonium formate aqueous solution containing 0.1% ammonia solution, and mobile phase B was methanol. The gradient elution program was started at 80% A for 1 min, dropped to 35% A in 1 min, decreased to 20% A in 2 min, held for 5 min, decreased to 10% A within 2 min, held for 3 min, resumed at 80% A within 1 min, and kept constant for 5 min. Figure 2 shows the typically extracted ion chromatograms of the mixed standard solution of peimisine, sipeimine, peiminine, and peimine at the concentration of 0.5 µg/mL.

**Figure 3:**
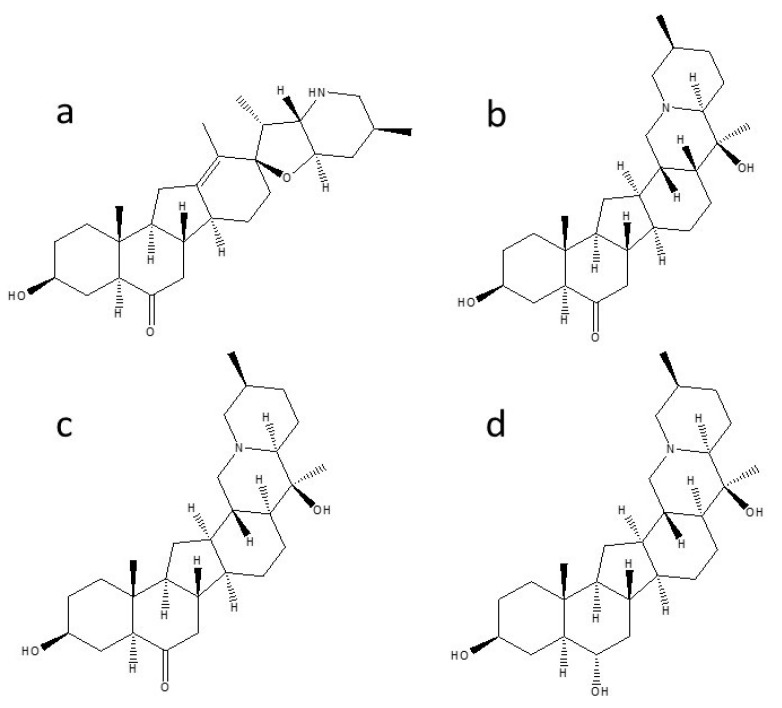
Chemical structures of four isosteroidal alkaloids: (**a**) Peimisine, (**b**) Sipeimine, (**c**) Peimine, (**d**) Peiminine.

The mass spectrometer was equipped with an electrospray ionization (ESI) interface operating in positive ion mode. The selected reaction monitoring (SRM) mode was used to acquire the mass spectrometric data. The full width at half maximum (FWHM) of Q1 and Q3 was 0.7 for both. The optimal ESI source parameters were set as follows: the capillary temperature was 300 °C; spray voltage was 4600 V; the pressure of sheath gas, aux gas, and ion sweep gas was maintained at 45, 10, and 10 arb units, respectively. The ion transitions and optimal collision energy of selected reaction monitoring chosen for quantitative analysis were as follows: m/z 428→m/z 114 (44 eV) for peimisine; m/z 430→m/z 138 (48 eV) for sipeimine; m/z 430→m/z 412 (38 eV) for peiminine; m/z 432→m/z 414 (40 eV) for peimine. The retention time, protonated molecule ions (represented as [M+H]+), and the analytical parameters of the developed method for analysis of four alkaloids are listed in Table 1, and chemical structures are shown in Figure 3. 

### 2.8. Statistical Analysis

Software SAS 9.1 was used for statistical analysis. Data were subjected to the least significant difference (LSD) test at a 5% probability level (*p* < 0.05) wherever possible. In Table 2 and Table 3, the number of replicates is 3 (three bottles under each LED light treatment). In Table 4, the number of replicates is 21 (each bottle had 7 explants, and each treatment had 3 bottles). The experiments were repeated three times, including LC-MS/MS analysis.

## 3. Results and Discussion

### 3.1. Callus Proliferation

**Figure 4:**
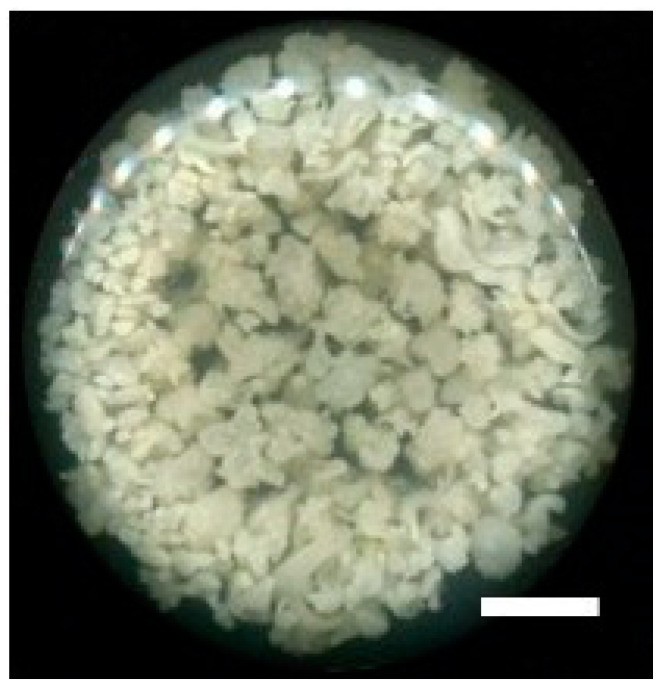
Embryogenic calli (EC) of *F. cirrhosa* D. Don growing in Murashige and Skoog’s liquid basal medium supplemented with 2,4-D (0.5 mg/mL) and 2% sucrose. The image is a photograph taken by a scanner of the bottom of the culture flask after six weeks of incubation (bar = 1.2 cm).

Callus of *F. cirrhosa* cultured in Murashige and Skoog’s liquid medium with 2,4-D, under agitated conditions in dark incubation for six weeks, proliferated readily. Callus not only grew in volume but also became embryogenic since early stages of embryos were observed (Figure 4). There are several reports from our laboratory where various secondary metabolites have been obtained from different culture systems [25], including callus cultures of several medicinally important plant species, e.g., *Salvia miltiorrhiza* Bunge [26] and *Saussurea involucrata* Kar. et Kir. [27]. These and numerous other reports demonstrate that callus cultures have tremendous potential for the sustainable and large-scale production of secondary metabolites used in pharmaceuticals. Biotechnological applications of plant callus cultures have been recently reviewed [28], and, according to the author, the full potential of callus plant culture technology has not yet been exploited. Callus cultures and suspension cell cultures offer a wide range of applications in agriculture and horticulture, including for Chinese medicinal plants. Genetically transformed callus cultures cannot only be used for the synthesis of bioactive compounds but also for the development of plants with traits [28].

### 3.2. Influence of LED Lights on Morphogenesis of Embryogenic Calli (EC) of F. cirrhosa 

**Figure 5:**
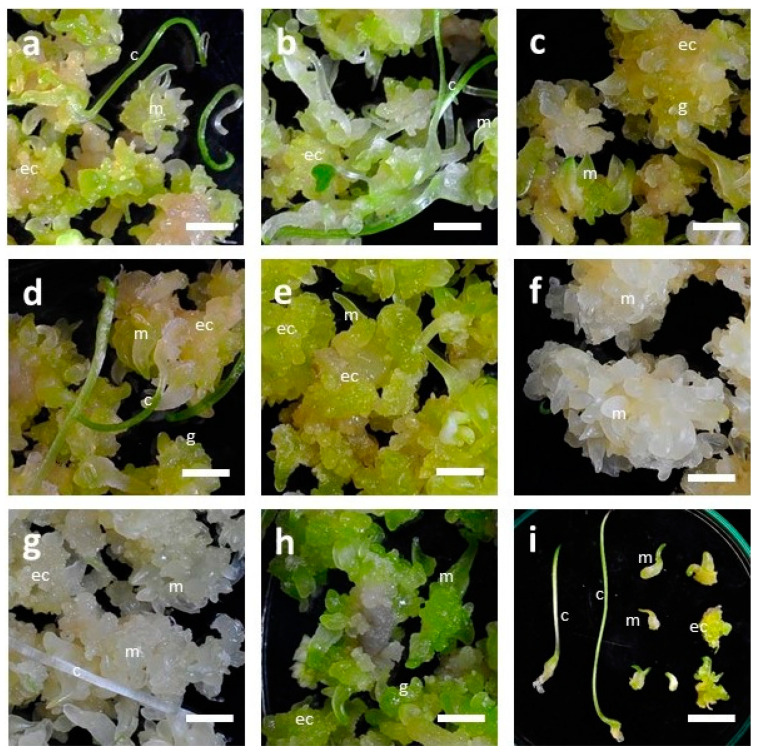
Influence of different LED lights on the growth and development of embryogenic calli of *F. cirrhosa* D. Don: (**a**) CW-5000K; (**b**) WW-2700K; (**c**) 7R1G1B; (**d**) 8R1B; (**e**) 9B; (**f**) 9R; (**g**) 9IR; (**h**) 3R3B3IR; (**i**) Different stages of somatic embryos. For microphotographs, cultures are taken out of the bottles and transferred to sterilized Petri dishes. g: globular embryos; m: mature embryos; c: cotyledonary leaf; ec: embryogenic callus. (a–f, h, bar = 0.57 cm; g, bar = 1.4 cm). Culture medium is Murashige and Skoog’s basal medium supplemented with 2% sucrose and 0.4% GPP. Culture vessels are 650 mL glass bottles, each containing 100 mL medium. Observations recorded after three months of incubation in a specially designed LED light growth chamber.

The eight LED lights had significant effects on the growth and development of embryogenic calli (EC) of *F. cirrhosa* (Table 2). Although the development of somatic embryos (SEs) in embryogenic calli were recorded under all the light treatments, the maximum number of SEs was recorded under red (9R, 223.7), infrared (9IR, 231.3), and a combination of red+blue+infrared light (3R3B3IR, 230.7), respectively. Among the red, infrared, and a combination of red+blue+infrared LED lights, there was a low significant difference concerning the number of SEs and the number of SEs with cotyledonary leaves (Table 2). LED lights also influenced the number of embryos with the development of cotyledonary leaves. Calli exposed to white light (WW-2700K) developed a maximum number of SEs with cotyledonary leaves (22.3), followed by 8R1B (16.7) and CW-5000K (12.0), respectively. SEs under LED lights 9B, 9R, 9IR, and 3R3B3IR developed a reduced number of cotyledonary leaves (in the range of 3.7 to 5.3). There was a low significant difference in the total fresh weight of EC among different LED light treatments. The maximum total fresh weights (FW) of EC biomass, i.e., 17.92 g and 16.67 g, were recorded with treatments of red (9R) and a combination of red+blue+infrared light (3R3B3IR), respectively. Eight LED lights also influenced the growth and development, and morphological features of somatic embryos. Embryogenic calli exposed to different LED lights developed somatic embryos in different developmental stages from early globular to mature SEs with cotyledonary leaves (Figure 5a–i). However, the average number of SEs varied depending upon the LED light. LED light WW-2700K developed the maximum number of SEs with cotyledonary leaves (22.3), followed by 8R1B (16.7) (Table 2). Different LED lights also affected the overall color of the embryogenic calli mass (Figure 5a–h). Under red (9R) and infrared (9IR) treatments, cultures turned white (Figure 5f,g), while under red+blue+infrared (3R3B3IR), these were dark green (Figure 5h). 

Light is one of the essential components required by plants for photosynthesis. However, its quantity and duration (photoperiod) drastically affect plant growth and development [29]. Fluorescent tubes are the most common lighting source in a culture room in a typical tissue culture set up. However, in the recent past, due to several advantages, more advanced light-emitting diodes (LEDs) have been used as a source of light. LEDs are relatively cool, emit light of specific wavelengths (spectra), are much smaller in size, and are more durable compared to conventional ones [30]. Due to their efficiency in growth and development, LED lights have been used for micropropagation of many horticultural and agricultural crops [31,32,33]. Depending on requirements, different types of growth chambers equipped with LED lights can be designed. Since the supply of specific light spectra can be controlled in plant tissue culture systems via LED lights, the effects of individual or combinations of light spectra on plant growth and development can be investigated appropriately as in the present study. Several studies on the influence of LED lighting on plant growth, physiology, and secondary metabolism have been reviewed [11,34]. Recently, Pedmale and co-workers reported that the quality of light affects plant growth and development by the regulation of different mechanisms, including the selective activation of light receptors, such as phytochromes by red and far-red light, cryptochromes and phototropins by blue light, and UV-B receptors by ultraviolet light [35]. In a previous study in our laboratory, LED lights affected the development of somatic embryos and callus proliferation (fresh weight) in *Peucedanum japonicum* Thunb [22]. Several studies have demonstrated that the quality, duration, and intensity of red, infrared, blue, and ultraviolet light can have a profound influence on plants by activating or deactivating physiological reactions and controlling their growth and development [36,37,38]. These studies confirm with many other reports that LED lights are more efficient in plant growth compared to fluorescent lamps. Similar to our results, the beneficial effects of some LED light sources on the induction of embryogenesis in *Oncidium* have been reported [39]. Due to these beneficial effects, LED light systems are being increasingly used to boost the horticulture industry in Taiwan and several other countries [40,41,42].

### 3.3. Influence of LED Lights on the Contents of Isosteroidal Alkaloids in In Vitro Cultures of F. cirrhosa 

Responses of LED lights drastically varied among the four alkaloids. Out of four alkaloids tested (by LC-MS/MS) in in vitro cultures of *F. cirrhosa,* the most noticeable effects of eight LED lights were recorded in the case of peiminine (Table 3). The maximum peiminine in cultures (2.40 µg/g/dw) was detected under red (9R) light, followed by infrared (9IR) (2.21 µg/g/dw). Peiminine content in cultures exposed to LED lights CW-5000K, WW-2700K, 7R1G1B, and 3R3B3IR was in the range of (0.12–0.28 µg/g/dw). In in vitro-derived bulblets (3 months old), peiminine content (2.98 µg/g/dw) was detected in the sample grown under fluorescent light. However, this alkaloid could not be detected in cultures exposed to LED lights with 8R1B or in commercial bulbs (3 years old, wild type). Alkaloid sipeimine could not be detected in any in vitro culture or under any light treatment. The only material in which sipeimine in low quantities (0.6 µg/g/dw) could be detected was commercial bulbs (Table 3). Contents of another alkaloid peimisine in cultures were recorded only under two LED light treatments, 9R (3.65 µg/g/dw) followed by 9IR (3.22 µg/g/dw). Between two types of bulbs, the maximum peimisine was noted in commercial bulbs (68.4 µg/g/dw), followed by in vitro-derived bulblets (0.91 µg/g/dw). Similar to peimisine, the presence of peimine was found only under two LED light treatments, 9R (0.38 µg/g/dw) and 9IR (0.05 µg/g/dw), though the quantities were much lower compared to peimisine. Like peiminine, peimine was not detected in commercial bulbs (wild types). 

Light quality affects the photochemical control of gene expression in various metabolic pathways, affecting the synthesis of nucleic acids, amino acids, organic acids, and sugars, etc., which are essential not only for cell growth and development but also for cell maintenance [43,44]. The response of plant cells to stress and their reorientation to developmental programs results in the expression of protein kinases, transcription factors, and structural genes that contribute to the adaptation [45]. Similar to the present study, the beneficial effects of LED lights on the contents of bioactive compounds in *Peucedanum japonicum* Thunb have been reported in our laboratory [22]. 

### 3.4. Development of Bulblets in Somatic Embryos of F. cirrhosa 

**Figure 6:**
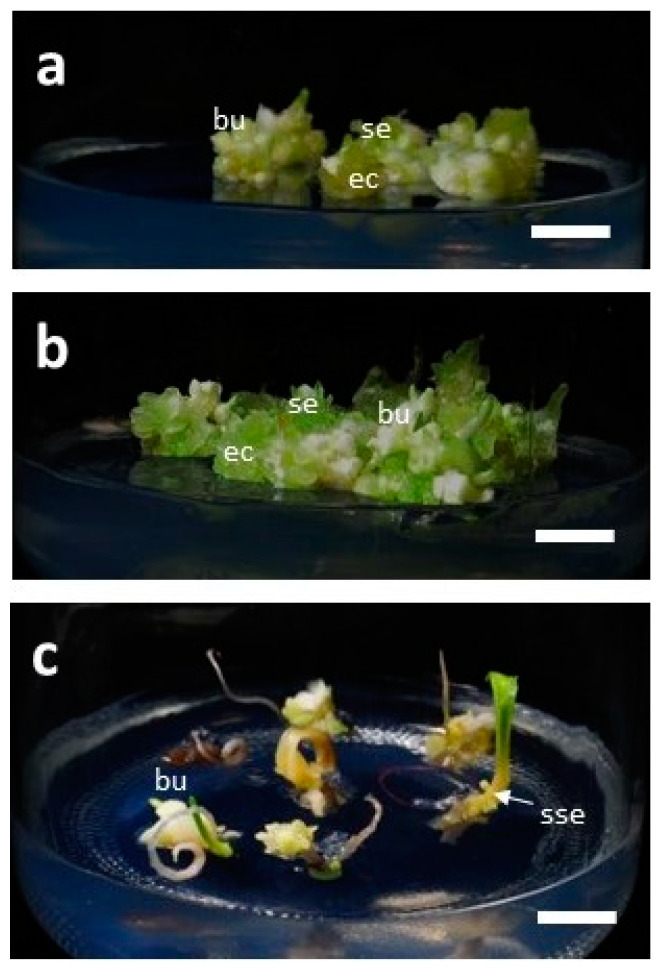
Development of bulblets in somatic embryos of *F. cirrhosa* D. Don (photographs taken after three months of incubation): (**a**) Bulblets from a single embryo, (**b**) bulblets in a cluster of five embryos, (**c**) bulblets in embryo with cotyledon leaf. bu: bulblet; se: somatic embryo; sse: secondary somatic embryo; ec: embryogenic callus. Bar = 0.8 cm.

**Figure 7:**
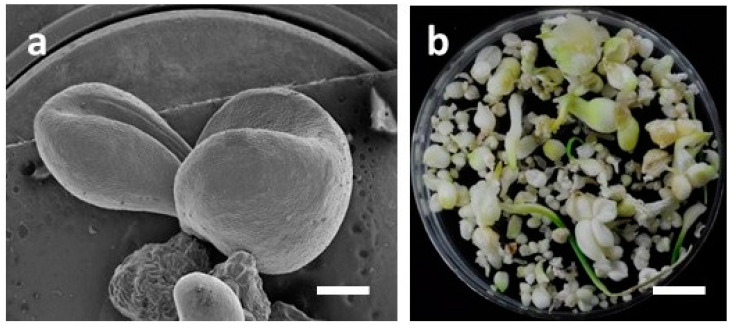
Development of bulblets from somatic embryos of *F. cirrhosa* D. Don: (**a**) Scanning electron microscopy (SEM) of bulblets (bar = 0.77 mm), (**b**) different stages of growth of culture-derived bulblets of *F. cirrhosa* (bar = 1.0 cm).

Bulblet formation was observed in all three types of somatic embryos taken from cultures under LED lights CW5000K or WW2700K, as described in Section 2.3 (Table 4; Figure 6a–c; Figure 7a–b). However, the response percentage and number of bulblets varied depending upon the developmental stage of the somatic embryos. The highest response of bulblet formation (90%) was recorded with single embryos with an average of 4.7 bulblets/SE. In the case of a cluster of five embryos, the percentage of response and the average number of bulblets were 86.7 and 3.3 bulblets/SE, respectively. The lowest response (43.6%) and the least number of bulblets (1.1/SE) were recorded in SEs with cotyledonary leaf. However, in this case, some secondary somatic embryos at the base of cotyledonary leaf were observed, though cotyledonary leaf did not grow further, and withered and dried. Single embryos grew further in size and developed multiple bulblets without much callus growth. The cluster of five embryos grew in size and developed further embryogenic callus and secondary somatic embryos. 

Since bulbs constitute the most critical parts of *Fritillaria* plants and are the primary source of isosteroidal alkaloids in *Fritillaria* species used in traditional Chinese medicine (TCM), the production of bulbs in *F. cirrhosa* by tissue culture technology is highly desirable. The objective of the present study was to investigate the effects of LED lights on the growth and development of embryogenic callus and the analysis of alkaloid contents in cultures; however, the development of bulblets in somatic embryos is an important observation in the study because in natural conditions, one bulb typically develops into a single seedling and it takes about 5–6 years to grow into an appropriate size [46]. It has also been reported that isosteroidal alkaloids in *F. cirrhosa* bulbs are greatly influenced by environmental conditions, plant age, and harvest times [47]. Recently, Chang and co-workers in our laboratory reported an efficient micropropagation method of bulblet production in *F. cirrhosa* and also the presence of some isosteroidal alkaloids in tissue culture-derived bulblets and callus [10]. 

## 4. Conclusions

Eight LED lights influenced the growth and development, and morphological features of somatic embryos of *F. cirrhosa* D. Don. Embryogenic calli exposed to different LED lights developed somatic embryos in different developmental stages from early globular to mature SEs with cotyledonary leaves. The average number of somatic embryos that developed varied depending upon the wavelength of light emitted by the LEDs. The maximum number of SEs was recorded under red light spectra, followed by infrared and a combination of red/blue/infrared light spectra, respectively. Concerning alkaloids, the most significant effects were recorded with red and infrared light spectra in which peimisine, peimine, and peiminine were recorded. Sipeimine was not detected in any culture. Results obtained in the study indicate that red and infrared light spectra may be useful to obtain peimisine, peimine, and peiminine from callus cultures of *F. cirrhosa* D. Don. However, further research is needed to boost the quantities of these compounds in cultures and also for optimization to scale up production in bioreactors for commercial feasibility. Further research is also required for the optimization of large qualities of bulblet production in cultures. The production of important and precious alkaloids under a laboratory set up may reduce our dependence on natural materials from the wild and may help in the conservation of vulnerable plant species, such as *F. cirrhosa* D. Don. 

## Figures and Tables

**Table 1:** LC-MS/MS based method development and validation for four standards.

Marker Compounds	tR ^a^ (min)	Mass/Charge (m/z)	Linearity and Range			Sensitivity ^c^	
			Regression Equation ^b^	Correlation Coefficient (r^2^)	Linear Range (μg/g)	LOD (ng/g)	LOQ (ng/g)
Peimisine	5.78	428.316	y = 0.0396x + 0.0630	0.9923	0.1–40	0.01	0.04
Sipeimine	8.22	430.332	y = 0.5426x + 0.1531	0.9968	0.1–40	0.02	0.06
Peiminine	10.72	430.332	y = 0.0047x + 0.0010	0.9967	0.1–40	0.97	3.23
Peimine	11.88	432.347	y = 0.0067x + 0.0016	0.9977	0.1–40	0.56	1.88

^a^ tR: Retention time. ^b^ The regression equations are presented as y = mx+c, and y and x are defined as peak area and concentration of the compound, respectively. ^c^ LOD: Limit of detection, S/N = 3; LOQ: limit of quantification, S/N = 10.

**Table 2:** Influence of different LED lights on the growth and development of embryogenic callus cultures *.

LED Light Treatment	Av No. of Somatic Embryos/Bottle	Av No. Of Somatic Embryos with Cotyledonary Leaves/Bottle	Av Total Fresh Weight of Cultures/Bottle (g)	Morphological Features of Cultures (Somatic Embryos = SEs)
CW-5000K *	63.7 ± 7.6 ^c^ **	12.0 ± 6.0 ^abc^	12.82 ± 1.09 ^c^	SEs at all stages from globular to mature, SEs with long cotyledonary leaves. Color of cultures is light green.
WW-2700K	122.0 ± 65.0 ^bc^	22.3 ± 13.9 ^a^	13.64 ± 2.82 ^c^	SE stages are similar to CW-5000K. Color of cultures is light green.
7R1G1B	108.0 ± 38.2 ^bc^	8.5 ± 3.5 ^bc^	12.99 ± 2.02 ^c^	A majority of SEs in early stages, including globular shapes. Cotyledonary leaves absent. Color of cultures is light green to light brown.
8R1B	157.3 ± 9.3 ^ab^	16.7 ± 8.3 ^ab^	15.23 ± 0.97 ^abc^	Stages similar to CW-5000K. Color of cultures is light green.
9B	169.0 ± 66.1 ^ab^	3.7 ± 2.3 ^c^	14.61 ± 1.02 ^bc^	SE stages similar to CW-5000K, but cotyledonary leaves shorter in length. Color of cultures is light green to light yellow.
9R	223.7 ± 57.5 ^a^	5.3 ± 4.9 ^c^	17.92 ± 0.77 ^a^	A majority of SEs in the early stages, including globular shapes. Cotyledonary leaves absent. Color of cultures is white.
9IR	231.3 ± 62.3 ^a^	4.7 ± 0.6 ^c^	16.67 ± 1.85 ^ab^	SE stages and color of cultures similar to 9R. Only a few cotyledonary leaves are seen. Color of cultures is white.
3R3B3IR	230.7 ± 23.4 ^a^	4.3 ± 2.5 ^c^	17.56 ± 2.35 ^a^	SE at all stages from globular to mature but cotyledonary leaves shorter in length. Color of cultures is dark green.

* Murashige and Skoog’s basal medium supplemented with 2% sucrose and 0.4% GPP. pH = 5.7 ± 0.1. Observations recorded after three months of incubation. ** Mean ± standard error. Means within each column followed by the same letter(s) are not significantly different at 5% level by Fisher’s protected LSD test.

**Table 3:** LC-MS/MS analysis of four isosteroidal alkaloids in in vitro cultures exposed to eight different LED lights, in vitro derived bulblets (3 months old), and commercial *Fritillaria cirrhosa* D. Don bulbs (wild type, three years old).

LED Light Treatment	Plant Material		Isosteroidal Alkaloids (µg/g/dw)			Total of Four Alkaloids (µg/g/dw)
		Peimisine	Sipeimine	Peimine	Peiminine	
CW-5000K	In vitro cultures	ND *	ND	ND	0.12 ± 0.20 ^b^ **	0.12 ± 0.20 ^c^ **
WW-2700K	In vitro cultures	ND	ND	ND	0.28 ± 0.27 ^b^	0.28 ± 0.27 ^c^
7R1G1B	In vitro cultures	ND	ND	ND	0.19 ± 0.32 ^b^	0.19 ± 0.32 ^c^
8R1B	In vitro cultures	ND	ND	ND	ND	0.00 ± 0.00 ^c^
9B	In vitro cultures	ND	ND	ND	0.60 ± 0.43 ^b^	0.65 ± 0.45 ^c^
9R	In vitro cultures	3.65 ± 1.68 ^b^ **	ND	0.38 ± 0.11 ^a^	2.40 ± 0.30 ^a^	6.42 ± 2.06 ^b^
9IR	In vitro cultures	3.22 ± 3.28 ^b^	ND	0.05 ± 0.09 ^b^	2.21 ± 0.87 ^a^	5.48 ± 3.21 ^b^
3R3B3IR	In vitro cultures	ND	ND	ND	0.26 ± 0.24 ^b^	0.26 ± 0.24 ^c^
Fluorescent tube	In vitro bulblets (3 months old)	0.91 ± 0.97 ^b^	ND	ND	2.98 ± 1.09 ^a^	3.90 ± 1.51 ^bc^
Natural habitat	Commercial bulbs (wild type, 3 years old)	68.4 ± 7.8 ^a^	0.6 ± 0.4 ^a^	ND	ND	69.0 ± 7.4 ^a^

* ND: Not detected. ** Mean ± standard error. Means within each column followed by the same letter(s) are not significantly different at 5% level by Fisher’s protected LSD test.

**Table 4:** Development of bulblets in single embryo, a cluster of five embryos, and single embryo with cotyledonary leaf (3–6 cm long) in *Fritillaria cirrhosa* D. Don.

Type of Somatic Embryo (SE) *	Percentage of Response (%)	Av No. of Bulblets/SE
Single embryo	90.0 ± 10.0 ^a^ **	4.7 ± 1.3 ^a^ **
Cluster of five embryos	86.7 ± 12.0 ^a^	3.3 ± 1.5 ^ab^
Embryo with the cotyledonary leaf	43.6 ± 29.0 ^b^	1.1 ± 0.7 ^b^

* Culture medium MSBM supplemented with 2% sucrose, 0.4% GPP. Observations recorded after three months of incubation. ** Mean ± standard error. Means within each column followed by the same letter(s) are not significantly different at 5% level by Fisher’s protected LSD test.
